# The VALTIVE1 study protocol: a study for the validation of Tie2 as the first tumour vascular response biomarker for VEGF inhibitors

**DOI:** 10.1186/s12885-024-13073-0

**Published:** 2024-10-24

**Authors:** Margherita Carucci, Andrew Clamp, Cong Zhou, Chris Hurt, Rosalind Glasspool, Phillip J. Monaghan, Sally Thirkettle, Michael Wheatley, Madia Mahmood, Monica Narasimham, Tracy Cox, Hilary Morrison, Susan Campbell, Annmarie Nelson, Daniella Holland-Hart, Noreen Hopewell-Kelly, Abin Thomas, Catharine Porter, Magdalena Slusarczyk, Alys Irving, Caroline Dive, Richard Adams, Gordon C. Jayson

**Affiliations:** 1 Centre for Trials Research, Cardiff University, 6thFloor, Neuadd Meirionnydd, Heath Park, Cardiff, CF14 4YS UK; 2 The University of Manchester, The Christie NHS Foundation Trust, Manchester, UK; 3 Cancer Research National Biomarker Centre, The University of Manchester, Manchester, UK; 4 University of Southampton, Southampton, UK; 5 Beatson West of Scotland Cancer Centre and University of Glasgow, Glasgow, UK; 6 The Christie Pathology Partnership, Manchester, UK; 7 The University of Manchester, Manchester, UK; 8 The Christie NHS Foundation Trust, Manchester, UK; 9 Centre for Trials Research, Cardiff University, Cardiff, UK; 10 Marie Curie Research Centre, Cardiff University, Cardiff, UK

**Keywords:** Clinical trial, Ovarian cancer, VEGFi, Tie2, Clinical decision making

## Abstract

**Background:**

Anti-angiogenic, VEGF inhibitors (VEGFi) increase progression-free survival (PFS) and, in some cases, overall survival in many solid tumours. However, their use has been compromised by a lack of informative biomarkers. We have shown that plasma Tie2 is the first tumour vascular response biomarker for VEGFi in ovarian, colorectal and gall bladder cancer: If plasma Tie2 concentrations do not change after 9 weeks of treatment with a VEGFi, the patient does not benefit, whereas a confirmed reduction of at least 10% plasma Tie2 defines a vascular response with a hazard ratio (HR) for PFS of 0.56. The aim of the VALTIVE1 study is to validate the utility of plasma Tie2 as a vascular response biomarker and to optimise the Tie2-definition of vascular response so that the subsequent randomised discontinuation VALTIVE2 study can be powered optimally.

**Methods:**

VALTIVE1 is a multi-centre, single arm, non-interventional biomarker study, with a sample size of 205 participants (176 bevacizumab-treated participants + 29 participants receiving bevacizumab and olaparib/PARPi), who are 16 years or older, have FIGO stage IIIc/IV ovarian cancer on treatment with first-line platinum-based chemotherapy and bevacizumab. Their blood plasma samples will be collected before, during, and after treatment and the concentration of Tie2 will be determined. The primary objective is to define the PFS difference between Tie2-defined vascular responders and Tie2-defined vascular non-responders in patients receiving bevacizumab for high-risk Ovarian Cancer. Secondary objectives include defining the relationship between Tie2-defined vascular progression and disease progression assessed according to RECIST 1.1 criteria and assessing the impact of PARPi on the plasma concentration of Tie2 and, therefore, the decision-making utility of Tie2 as a vascular response biomarker for bevacizumab during combined bevacizumab-PARPi maintenance.

**Discussion:**

There is an urgent need to establish a test that tells patients and their doctors when VEGFi are working and when they stop working. The data generated from this study will be used to design a second trial aiming to prove conclusively the value of the Tie2 test.

**Trial registration:**

ClinicalTrials.gov identifier: NCT04523116. Registered on 21 Aug 2020.

## Background

Angiogenesis has been validated as a target in multiple clinical trials that have compared conventional therapy to the same regimen supplemented by anti-angiogenic Vascular Endothelial Growth Factor inhibitors (VEGFi) [1–3]. In many tumour types, these studies have shown that VEGFi improve Progression Free Survival (PFS) and, in some cases, Overall Survival (OS). In relation to ovarian cancer (OC), studies have shown that VEGFi as bevacizumab improve PFS in the first, [4–6] second [7, 8] and subsequent [9, 10] lines of treatment, irrespective of the platinum-sensitivity of the disease. Importantly, recent data have shown that use/re-use of bevacizumab is clinically effective [11], introducing the concept of recurrent use of VEGFi for maintenance after each course of cytotoxic chemotherapy for OC. Clearly, cost-effective use and re-use mandate the development of biomarkers that will optimise use of VEGFi.

### First biomarker for VEGF inhibitors

We have carried out biomarker discovery studies in ovarian (OC) [12, 13] and colorectal (CRC) [14] cancer clinical trials, in which patients were treated with cytotoxic agents and bevacizumab. For the first time we have shown in both data sets that a reduction in plasma Tie2 concentration on-treatment is a vascular response biomarker for bevacizumab, where reduction in its plasma levels is a surrogate for vascular response and its subsequent elevation represents anti-angiogenic resistance, a biologically plausible finding given the relationship between VEGF and Ang/Tie2 pathways [15–17]. In the CRC trial, MRI data demonstrated that the plasma Tie2 signal represents changes in tumour vasculature during treatment that reflect acute and medium-term reductions in tumour volumes [14]. Modelling the vascular Tie2 data in conjunction with epithelial cell biomarker data (CA-125 and CK18, for OC and CRC, respectively) showed that we could predict progressive disease significantly better if both tissue compartments, vascular and epithelial, were modelled together confirming the clinical significance of Tie2 in both diseases. Thus, we have identified for the first time a minimally invasive tumour vascular response biomarker for bevacizumab. We have previously demonstrate that this is not an idiosyncratic finding from a single laboratory and have demonstrated a drug targeted class effect. This is evidenced by the same pharmacodynamic changes in Tie2 being reported in different laboratories that have analysed samples taken from patients with glioma [18], CRC [19] and gall bladder [20] cancer, who were treated with cediranib, a VEGF receptor Tyrosine Kinase inhibitor (VEGFR TKi). Together these data suggest that Tie2 is a generic tumour vascular response biomarker for VEGFi.

We have also analysed the trajectory of plasma Tie2 concentrations in the ABC03 trial, a randomised trial of cytotoxic chemotherapy with or without cediranib in biliary tract cancer [20, 21]. Plasma Tie2 concentrations rose slightly in patients treated with cytotoxic chemotherapy but significantly reduced (*p* = 0.004) in patients who received cediranib, in a manner that resembled our findings in OC/CRC patients treated with bevacizumab. This indicates that plasma Tie2 is a generic vascular response biomarker for small and large molecule VEGFi. Tie2 defined vascular responders demonstrated an improved PFS HR of 0.56 compared to vascular non-responders, which was significant in univariate analysis (*p* = 0.034) and marginally significant in multivariate analysis (*p* = 0.054). However, the proportion of patients without a Tie2 response (32%) was greater than that seen in our OC and CRC studies (25%), and vascular non-responders demonstrated shorter PFS (HR = 1.35, *p* = 0.28) than the patients treated with cytotoxic chemotherapy alone. This suggests that the toxicity from cediranib negatively impacts on the amount of chemotherapy patients could tolerate, as previously reported [20, 21]. This then explains why the trial did not detect a treatment benefit from cediranib (HR = 0.93, *p* = 0.72). We already showed an HR of 0.56 for progression in favour of plasma Tie2 responders in bevacizumab-treated CRC patients [14]. We have added to this analysis another cohort of 37 patients who were treated with cytotoxic chemotherapy only [22]. Plasma Tie2 concentrations did not change in patients receiving cytotoxic therapy only and in a multi-variate analysis that took PS, stage and LDH into account, the HR for PFS in favour of Tie2 responders was 0.54 (*p* = 0.039) whereas the HR for bevacizumab-treated Tie2 non-responders resembled the control group i.e. there was no benefit from bevacizumab in the Tie2 non-responding group. Together the two newly analysed datasets augment our publications and show that Tie2-defined vascular non-responders do not benefit from VEGFi. It is this observation that underpins the strategy behind VALTIVE1 and 2; namely, that we aim to identify Tie2-defined non-responders so that ineffective VEGFi treatment can be stopped early minimising toxicity and intravenous therapy while optimising cost effectiveness.

Our data show that plasma Tie2 is impacted by VEGFi but not cytotoxic chemotherapy [13]. Therefore, we can take blood samples for Tie2 analysis irrespective of whether VEGFi start concurrently with cytotoxic chemotherapy. The data also allow us to distinguish epithelial from vascular tissue compartment behaviour on an individual patient basis. The results show that bevacizumab is associated with a 75% Tie2-defined vascular response rate where a vascular response is defined as at least 10% reduction in Tie2, based on two or more measurements within 9 weeks of starting bevacizumab, compared with pre-treatment levels [14]. The other patients are deemed vascular non-responders. Tie2-defined vascular progression is observed in vascular responders when plasma Tie2 increases > 50% above the nadir concentration. As our principal strategy is to identify Tie2-non-responders, who do not appear to benefit from VEGFi, we will focus on identifying the 25% of patients whose Tie2 reduces by less than 10% over at least 3 measurements.

### Bevacizumab + PARPi

A further class of maintenance therapy for OC includes PARP inhibitors (PARPi) [23]. Several trials have shown that PARPi improve PFS in high grade serous ovarian cancer [24–27] and are more effective in the context of BRCA gene mutation (BRCAm) or Homologous Recombination Deficiency (HRD). However, these agents still improve PFS even in the absence of these markers [28]. In multiple clinical settings, VEGFi augment the response to co-administered cytotoxic chemotherapy [3] and this additivity has also been described for PARPi/VEGFi combinations [2, 29, 30]. The question is whether the interaction is just additive or synergistic, as hypothecated in several preclinical models [31].

Experimental evidence suggests that through direct signalling mechanisms (PDGFRα and VEGFR3) [32] and indirectly through the induction of hypoxia [33, 34], VEGFi increase BRCAm-like phenotypes in tumour cells [35], thereby enhancing the efficacy of PARPi [32]. Conversely, knockdown of PARP-1 reduces expression of VEGF mRNA and microvessel density in vitro and tumour size in vivo [36], while an experimental PARPi inhibited several angiogenesis-related phenotypes in vitro [23]. Taking all of these data together there is pre-clinical evidence for a possible synergistic relationship between PARPi and VEGFi. If such a relationship were to exist, we would expect it to distort the Tie2-defined vascular response to VEGFi. However, the data pointing to synergy have emerged from experimental studies that have not been replicated or validated in the clinic. Thus, VALTIVE1 will test whether the Tie2 vascular signature is impacted by the sequential introduction of bevacizumab followed by PARPi olaparib. Despite pre-clinical data that support potential synergy and clinical data that point to additivity, our hypothesis is that the Tie2 vascular signature will not be impacted by olaparib, as olaparib is a biologically targeted cytotoxic agent and, to date, no cytotoxic drug has impacted on Tie2 in the clinic. These include carboplatin and paclitaxel in the first line treatment of ovarian cancer (TRICON7) [13]; FOLFOX in colorectal cancer (TRAVASTIN) [14]; cisplatin and gemcitabine in biliary tract cancer (ABC03) [21] and weekly paclitaxel in recurrent, platinum-resistant ovarian cancer (OCTOPUS – unpublished). Taking all of this information together, pre-clinical data suggest that there may be a synergistic relationship between olaparib and VEGF inhibitors. However, these mechanisms have not been seen in the clinic and as we have not seen any cytotoxic drug impact on Tie2, the cytotoxic effects of olaparib are unlikely to do so either. This lack of clarity concerning the PARPi-VEGFi relationship in HRD ovarian cancer underpins the rationale for an investigation on the change in plasma Tie2 concentrations after addition of olaparib maintenance.

### Rationale for the VALTIVE1 study

We are not using VEGFi in ovarian cancer (or other solid tumours) appropriately and it is therefore important to validate Tie2 for clinical decision making around the use/reuse of VEGFi. Successful implementation of Tie2 as a vascular response biomarker would improve the cost-effectiveness of the drug, allowing us to use it in a biologically and clinically optimised manner in the 1st line setting and more than once in patients who are benefiting while reducing the toxicity for patients where the drug is ineffective.

The aim of the VALTIVE programme is to carry out a non-intervention biomarker cohort study that will further validate the utility of plasma Tie2 as a vascular response biomarker but which will critically allow us to optimise the Tie2-definition of vascular response so that VALTIVE2, a randomised discontinuation study, can be powered optimally.

## Methods/design

### Study design

**Fig. 1:**
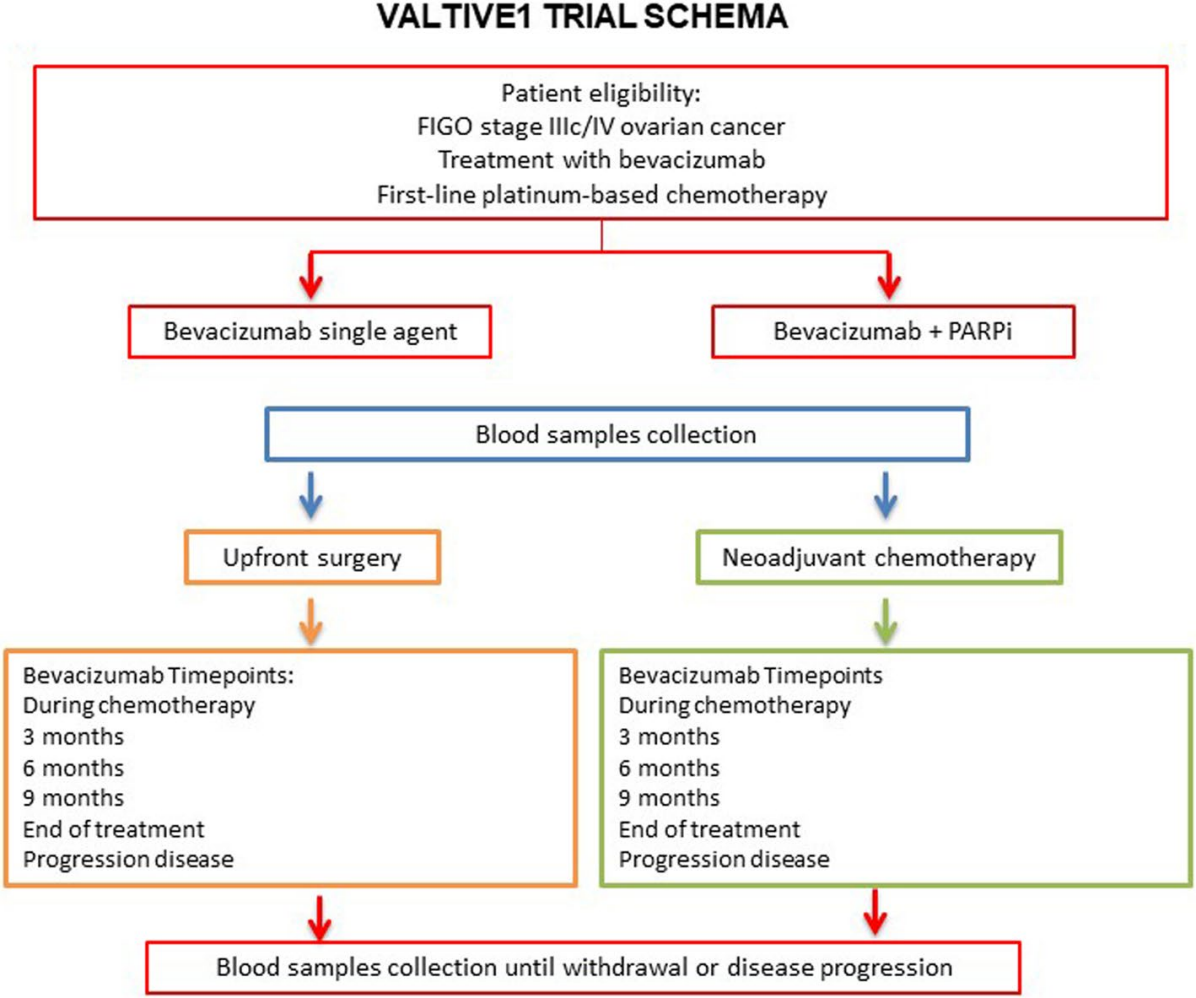
VALTIVE1 Study Schema

VALTIVE1 is a multi-centre, single arm, non-interventional biomarker study, which will enrol 176 bevacizumab-treated participants and 29 participants receiving bevacizumab and olaparib/PARPi, who are 16 years or older, have FIGO stage IIIc/IV ovarian cancer on treatment with first-line platinum-based chemotherapy and bevacizumab, and consent to the collection of their blood samples before, during, and after treatment (Fig. 1: VALTIVE1 Study Schema). Potential participants will be identified in their treating hospital and enrolled in one of the 20 secondary care hospitals in UK participating to the study as recruiting centres.

The study started recruitment on 31 March 2021 and the estimated study completion date is October 2025.

### Study objectives and outcome measures

#### Primary objective

To define the Progression Free Survival (PFS) advantage of Tie2-defined vascular responders vs Tie2-defined vascular non-responders in patients receiving bevacizumab for high risk Ovarian Cancer.

#### Secondary objectives

- ➢ To define the relationship between Tie2-defined vascular progression and disease progression assessed according to RECIST v. 1.1 criteria
- ➢ To assess the impact of PARPi on the plasma concentration of Tie2 and, therefore, the decision-making utility of Tie2 as a vascular response biomarker for bevacizumab during combined bevacizumab-PARPi maintenance

#### Exploratory objectives

- ➢ To optimise the number of samples and timelines of samples needed to adequately power the subsequent randomised discontinuation study
- ➢ To optimise the response algorithm for the randomised discontinuation study
- ➢ To optimise the threshold for Tie2-defined vascular progression as a predictor of disease progression according to RECIST v.1.1 criteria
- ➢ To engage patients in VALTIVE1 to best inform informed consent procedures in VALTIVE2 including patients’ attitudes towards randomised discontinuation studies
- ➢ To investigate the impact of surgery on the plasma concentrations of Tie2 in patients who do or do not start bevacizumab before surgery
- ➢ To investigate whether macrophages are responsible for the increase in Tie2 at the point of Tie2-defined vascular progression

### Study outcomes measures

#### Primary outcome measure

The primary outcome measure (associated with the primary objective) is to define the progression free survival (PFS) of Tie2-defined vascular responders and Tie2-defined vascular non-responders.

#### Secondary outcomes measures

- ➢ To optimise the definition of Tie2-defined vascular response in relation to RECIST1.1 or clinically-defined PFS
- ➢ To optimise the definition of vascular progression in relation to RECIST1.1 or clinically defined progressive disease
- ➢ To define the optimum number of samples and schedule of sample acquisition that will provide information on Tie2-defined response and progression
- ➢ To define the impact of surgery on plasma Tie2 concentrations
- ➢ To determine the acceptability of randomisation to Tie2-defined optimisation of bevacizumab
- ➢ To define the impact of PARPi on plasma Tie2 concentrations

### Eligibility criteria

#### Inclusion criteria

In order to be eligible for participation in this study, the patient must:

1. Be willing and able to provide written informed consent for the study
2. Age 16 years or over on day of signing informed consent
3. Histologically proven ovarian, primary peritoneal or fallopian tube cancer (henceforth referred to collectively as Ovarian Cancer—OC) that fulfils one of the following criteria:
  FIGO stage III with residual disease of more than 1 cm diameter after primary surgery (not required if the patient has Homologous Recombination Deficient OC and is considered eligible for combined bevacizumab with olaparib maintenance treatment or stage IV or
  stage III at presentation treated with neoadjuvant chemotherapy; or
  stage III with contraindication to debulking surgery chemotherapy

Note that additional eligibility criteria exist between the different devolved health administrations.

4. Planned to receive treatment with bevacizumab or biosimilar bevacizumab – bevacizumab must start by Cycle 5 of the cytotoxic chemotherapy regimen.
5. Be scheduled to receive at least 2 successive doses of bevacizumab with 6 or more weeks of follow up blood samples after the first dose of bevacizumab if given pre-operatively; or to start bevacizumab post-operatively
6. Be eligible for receiving treatment with first line, weekly or 3-weekly carboplatin and paclitaxel chemotherapy
7. Be willing to provide blood samples and comply with trial-specific procedures

#### Exclusion criteria

The patient must be excluded from participating in the study if the patient:

1. Is unsuitable for treatment with VEGF inhibitors
2. Is unable or unwilling to comply with study procedures
3. Is participating in a clinical study with an investigational product other than carboplatin, paclitaxel and bevacizumab
4. Is judged by the investigator to be unlikely to comply with study procedures
5. Is pregnant or could become pregnant and is not using adequate contraception
6. Has a known history of Human Immunodeficiency Virus (HIV) (HIV 1/2 antibodies)
7. Has known active Hepatitis B (e.g., HBsAg reactive) or Hepatitis C (e.g. HCV RNA is detected). Testing only required if patient has a history of either of these.

### Registration

Potential participants will be under the care of a consultant who specialises in the treatment of ovarian cancer. Once a patient has been identified as potentially eligible to participate, the opportunity will be discussed with the patient and she will be given a copy of the Participant Information Sheet (PIS) and Consent Form (CF). The patient will be given adequate time to consider the study and given the opportunity to ask further questions to the PI, their GP or other independent parties before deciding whether they wish to participate in the study. The participant must personally sign and date the current approved version of the CF before any study specific procedures are performed.

Consent will be taken by the Principal Investigator (PI) or a member of the study team who is GCP trained, suitably qualified and experienced, and who has been delegated by the PI to undertake this activity.

The right of the participant to refuse to participate in the study without giving reasons must be respected. The participant must remain free to withdraw at any time from the protocol treatment without giving reasons and without prejudicing her further treatment.

Eligible patients will be enrolled into VALTIVE1 as soon as they sign the Consent Form by the recruiting site staff using a web-based system.

### Study procedures

#### Screening and baseline assessments

All screening and baseline assessments should be performed within 14 days to determine the participant’s eligibility. The procedures undertaken and data recorded at the screening visit are:

- Informed consent
- Inclusion/exclusion criteria
- Full Physical Examination including vital signs, weight and height, and blood pressure
- Medical history
- Adverse Events
- Concomitant Medications: review of any medications received within 14 days before the start of treatment with bevacizumab
- Full Blood Count (FBC)
- Biochemistry profile
- CA-125
- Urinalysis
- Radiological assessment of disease by CT and reported according to RECIST v.1.1
- Blood plasma samples

#### Assessments on bevacizumab treatment

Participants will be treated according to local practice. Therefore, blood pressure and proteinuria will be managed according to standard local guidelines. Investigators should record:

- CA-125, each time it is measured, every 3 or 6 weeks
- Renal and hepatic function to be recorded at ± 3 weeks of the CT scans
- Blood plasma samples

#### Follow-up

After the end of treatment with bevacizumab, VALITVE1 Participants will be followed up until disease progression, withdrawal of consent, or death, whichever occurs first. The follow-up assessments to perform and record on CRF are:

- CA-125, each time it is measured, every 3 or 6 weeks
- Tumour imaging
- Blood plasma samples

If disease progression is recorded as per RECIST v.1.1 criteria, the participant will be requested to donate the last research blood sample.

#### Blood plasma samples collection

The definition of vascular response is either (i) stable or reduced concentrations of Tie2 after 6 weeks of treatment or (ii) a confirmed reduction in plasma Tie2 of > 10% within 9 weeks of starting treatment. Patients enrolled into the VALTIVE 1 study will provide blood samples twice before treatment with bevacizumab and then at the end of Cycles 1, 2, and 3, at the end of cytotoxic chemotherapy, at 3,6, and 9 weeks post cytotoxic chemotherapy, at 3 months, 6 months, 9 months, at the end of treatment, and at disease progression, resulting in a maximum of 14 samples per participant. The early samples will be used to refine the definition and number of required samples needed to define a Tie2-vascular response. The samples taken at 3, 6, and 9 weeks after cytotoxic chemotherapy will provide information on whether olaparib/PARPi impacts on the Tie2 vascular signature and how stable the Tie2 signature is in patients who remain on single agent maintenance bevacizumab. Samples collected at 3 months, 6 months, and 9 months post cytotoxic chemotherapy, at the end of treatment, and at disease progression will be used to investigate the relationship between Tie2-defined vascular progression and RECIST1.1 or clinically-defined progressive disease.

The sample acquisition schedule depends on whether the patient has upfront surgery or is entering the neoadjuvant pathway. If the patient is scheduled to receive neoadjuvant chemotherapy, the schedule of samples depends on which cycle she receives her first dose of bevacizumab and when surgery is scheduled. The principle is that two pre-treatment samples will be taken before the first dose of bevacizumab, with at least further samples being taken at the end of cycles 1 and 2 of bevacizumab. Where possible, a sample should be taken at the end of cycle 3 of bevacizumab, if this was not postponed by surgery.

Samples should preferably be taken on the day of treatment with bevacizumab, for instance through the cannula, and before drug administration. If this is not possible, samples should be taken as close to the time of treatment as possible.

#### Tumour Imaging assessments

Standard of care cross-sectional imaging of pelvis and abdomen will be performed and reported according to RECIST v.1.1 CT scan or other RECIST v.1.1 compatible imaging will be allowed, e.g. MRI, as long as the same imaging method is used throughout the study.

An assessment of baseline disease is required. For participants who have up front surgery before the treatment with bevacizumab, the baseline tumour imaging assessment will be performed 6 weeks ± 2 weeks post-operatively. For participants who receive neoadjuvant chemotherapy only and who subsequently do not have surgery or have an operation at which no resection occurs (an “open and shut” procedure), the pre-chemotherapy treatment, standard of care scan will be retrospectively reported to RECIST v.1.1 and will serve as the baseline scan. The baseline RECIST v.1.1 scan for all other participants will be conducted 6 weeks ± 2 weeks post-operatively, whether the participant has primary or delayed primary surgery (interval debulking surgery).

Further routine re-assessments imaging is required at the end of cytotoxic chemotherapy scan, during treatment with bevacizumab only if there are clinical symptoms of progression or there is CA-125 progression as defined by GCIG criteria, at the end of bevacizumab, and at disease progression unless this occurs before the end of bevacizumab, in which case scan at the end of bevacizumab would be omitted.

Progressive disease will be defined by the date of radiological or clinically identified progressive disease or death. CA-125 changes alone will not be sufficient to define progressive disease. Further, if an investigator considers that a patient is benefiting from bevacizumab, treatment until the conventional 12 months duration can continue even if RECIST1.1-defined progression has occurred.

#### Participant replacement

VALTIVE1 is critically dependent on acquisition of blood samples before and over at least 6 weeks after the first of two sequential doses of bevacizumab. Therefore, if a patient is recruited but does not receive two sequential doses of bevacizumab or provide two pre-treatment samples and further samples at the end of cycle 1 and cycle 2, the patient must be replaced. Nevertheless, she may continue to receive bevacizumab and can provide the other blood samples described in the protocol.

As of May 2021, patients with high grade ovarian cancer that have responded to first line surgery and chemotherapy may be eligible for maintenance therapy with niraparib, a PARP inhibitor that is given at the end of the platinum-based cytotoxic chemotherapy as a single agent (bevacizumab has to be stopped). In the case of participants who stop bevacizumab and start treatment with niraparib, sufficient blood samples are likely to have been collected to allow vascular response assessment before a patient changes to a PARP inhibitor. We therefore will retain at least 9 weeks of blood samples from such participants for vascular response assessment, follow them up for progression without taking extra blood samples, but replace them within VALTIVE1. Patients who change maintenance therapy to include PARP inhibitors should be managed according to local standard of care protocols.

### Sub-studies

#### Qualitative sub-study: participants experience

In order to optimise study design and recruitment to the potential randomised discontinuation trial (VALTIVE 2), a sample of VALTIVE 1 participants will be invited to participate in a qualitative sub-study. After signing the specific Qualitative sub-study Consent Form, participants will be interviewed by a qualified and experienced researcher with the aim to understand how patients feel about having the Tie2 biomarker test and how they might feel if their treatment was to be changed based upon the results from the test.

#### Translational sub-study: impact of surgery on Tie2

As surgery induces angiogenesis as part of wound healing, this sub-study will investigate the impact of surgery on plasma Tie2 concentrations and, more specifically, how Tie2-defined vascular response is impacted upon when an operation is performed as interval debulking surgery in patients who have received cytotoxic chemotherapy with or without bevacizumab, pre-operatively. Therefore, in 2 or 3 participating sites, up to 30 patients undergoing interval surgery will be invited to consent to the donation of extra plasma samples every week starting 2 weeks before surgery and extending to 4 weeks after surgery.

#### Translational sub-study: Tie2 and macrophage infiltration

Pre-clinical data and other biopsy data suggest that the increase in Tie2, seen at vascular progression, is related to macrophage infiltration with associated pro-angiogenic inflammation. This sub-study will analyse biopsies of amenable tissue from consenting participants at 1 or 2 participating sites with the aim to confirm this relation between Tie2 and macrophage infiltration.

#### Sample size

##### Arm: bevacizumab only

For the purposes of sample size calculation, survival data were simulated from the survival rate published in the ICON7 trial. Given a group of N simulated patients, with 75% vascular responders and 25% non-responders, the significance of survival difference between vascular responders and non-responders was estimated using a one-sided time dependent Cox-regression analysis, at a significance level of 0.05. The simulation was repeated 1000 times for each different value of N. We sought the minimum N value that would allow detection of significance in 800 simulation runs or more. We intend to recruit 176 patients (160 + 10% to allow for loss to follow up), of which 132 will have a Tie2-defined vascular response and 44 will be non-responders, to allow a statistical power of 80% and a significance level of 0.05 for the detection of an HR of 0.55. As the control arm of the ICON8b trial is the standard of care, we have agreement that up to 50 ICON8b control patients’ translational research samples and accompanying data would be available for this application. Therefore, in total we need to recruit at least 126 patients.

##### Arm: bevacizumab + PARPi

In this arm, we will test the hypothesis that olaparib has no impact on the expression level of Tie2 and, therefore, it does not compromise the decision-making utility of Tie2 as a vascular response biomarker for bevacizumab. Accordingly, the null hypothesis for the power calculation is that Tie2 trajectory will not be altered through the addition of olaparib. Based on our ICON7 data, we anticipate that Tie2 has a mean reduction of 0.04 and standard deviation of 0.19 (both on log_2_ scale) from 9 to 18 weeks (the estimated period during which olaparib would be added) in patients already receiving bevacizumab. Defining one standard deviation as the acceptance criteria for equivalence, we estimate that a minimum number of 23 participants will be needed to achieve a 90% power at a significance level of 0.05 based on a two one-sided t-test (TOST) for equivalence. We plan to recruit a total of 29 participants for this arm and the control cohort, allowing for 10% of patients who may develop early progressive disease and 15% of patients who may be lost to follow up during the study.

### Statistical analysis

The primary hypothesis is that in OC patients treated with bevacizumab for high-risk disease (CDF criteria) Tie2-defined vascular responders will have an improved HR for progression of 0.55 when compared with Tie2-defined non-responders. Time to progression will be calculated from date of start of carboplatin/paclitaxel chemotherapy to date of progression (as determined by RECIST v.1.1 or clinical assessment). Concentrations of plasma Tie2 will be measured at pre-defined time points prior to and during treatment. The biomarker data collected up to treatment cycle 3 will be used to categorise patients into vascular responders and non-responders. Here vascular responders are defined as patients with Tie2 reduction over 10% in repeated samples following treatment. A time dependent Cox regression analysis will be carried out to compare the PFS of patients with/without Tie2-defined vascular response. Clinical factors such as age, stage and other prognostic factors will be included in the analysis. The whole trajectory of Tie2 during treatment will be characterised using a Bayesian hierarchical modelling approach, as applied previously in our previous OC and CRC studies. The analysis aims to verify our observation in previous studies that Tie2 concentration will reduce following bevacizumab and will resume to pre-treatment levels by tumour progression in the vascular responding cohort. The model will be used to optimise the current criteria that define vascular response. We will seek answers to the following questions: (1) is a confirmed reduction of 10% at two time points sufficient to define vascular response? (2) are there alternative criteria if data were missing at one time point? (3) is the end of cycle 3 the optimum time to determine vascular response? (4) can we identify superior HR with more stringent definitions of Tie2-vascular response? (5) Will joint monitoring epithelial biomarker CA-125 and vascular biomarker Tie2 provide superior prediction of tumour progression. Once we have a validated definition of vascular non-response, we can move to design the randomised discontinuation non-inferiority trial VALTIVE2.

## Discussion

Until now, vascular responses have been defined through a mathematical approach based on the magnitude of VEGFi-induced Tie2 reduction and the intra- and inter-patient variation in Tie2 concentrations [21]. Consistent benefit in favour of vascular responders (average HR = 0.55) has been observed in the CRC and bile duct cancer (ABC03) datasets. Our aim in VALTIVE1 is to recruit sufficient patients to move beyond the mathematical definition of plasma Tie2 response and to develop clinically optimised definitions of vascular responders and non-responders by focusing on PFS. As a result, we envisage that VALTIVE2 will test for more significant HR in favour of vascular responders, increasing the clinical value of the Tie2 response assessment criteria in decision making around VEGFi treatment. In VALTIVE1, we will also explore the concept of partial vascular response, where Tie2 concentrations reduce but to a non-significant extent, i.e. Tie2 concentrations reduce by more than the 10% threshold that defines non-responders but less than the 30% definition of a complete vascular response.

VALTIVE1 is a biomarker study in which plasma samples will be taken before, during and following treatment of patients with ovarian cancer who are receiving bevacizumab. The aim of VALTIVE1 is to optimise the frequency and numbers of plasma samples being taken so that the definition of Tie2-vascular response is optimised. The study incorporates an extra set of samples after cytotoxic chemotherapy has been completed to monitor the Tie2 signature when olaparib or another PARPi is added to bevacizumab. A control set of samples will be taken in participants receiving single agent maintenance bevacizumab only to provide comparator data so that the impact of PARPi on an established Tie2 concentration–time curve can be determined. These data will then be used in a subsequent randomised study, VALTIVE2, in which patients receiving bevacizumab will be randomly allocated to the conventional 12 months of bevacizumab or Tie2-guided bevacizumab therapy.

## Data Availability

No datasets were generated or analysed during the current study.

## Abbreviations

CA-125: Cancer Antigen 125
CF: Consent Form
CRC: Colorectal Cancer
CRF: Case Report Form
CTR: Centre for Trials Research
FIGO: International Federation of Gynecology and Obstetrics
GCIG: Gynaecologic Cancer InterGroup
GCP: Good Clinical Practice
GP: General Practitioner
HCRW: Health and Care Research Wales
HIV: Human Immunodeficiency Virus
HR: Hazard Ratio
HRA: Health Research Authority
HRD: Homologous Recombination Deficiency
NHS: National Health Service
OC: Ovarian Cancer
PARPi: PARP inhibitor
PFS: Progression Free Survival
PI: Principal Investigator
PIS: Participant Information Sheet
RECIST: Response Evaluation Criteria In Solid Tumours
SOP: Standard Operating Procedure
VEGF: Vascular Endothelial Growth Factor
VEGFi: VEGF inhibitor
